# Indium-Based Silica Materials: Sustainable Syntheses Combined with a Challenging Insertion in SiO_2_ Mesoporous Structures

**DOI:** 10.3390/molecules29010102

**Published:** 2023-12-22

**Authors:** Amélie Maertens, Carmela Aprile

**Affiliations:** Laboratoire de Chimie des Matériaux Appliqués, Department of Chemistry, Namur Institute of Structured Matter (NISM), University of Namur, 5000 Namur, Belgium; amelie.maertens@unamur.be

**Keywords:** mesoporous silica materials, indium-based silica, silica nanospheres, acid-free SBA-15, green chemistry

## Abstract

Optimized sustainable procedures in both acidic and basic conditions are considered to meet some of the current environmental challenges of the scientific community. In this paper, the successful syntheses of two classes of indium-based silica nanomaterials are reported. Both procedures were conceived to enhance the sustainability of the synthesis methods and promote their preparations at room temperature while avoiding the hydrothermal treatment under static conditions at 100 °C. A fast, room-temperature synthesis of porous nanospheres was conceived together with an “acid-free” procedure for SBA-15-like materials. Moreover, the isomorphic substitution of silicon with indium was achieved. All the materials were deeply characterized to probe their structural, textural and morphological properties (e.g., transmission electron microscopy, N_2_ physisorption, ss MAS NMR of ^29^Si). The high specific surface area and the mesoporosity were always preserved even under the mild reaction conditions employed. The honeycomb structure and the spherical morphology of SBA-15-like materials and nanospheres, respectively, were also observed. The insertion of indium was confirmed via X-ray photoelectron spectroscopy (XPS) investigations.

## 1. Introduction

The interest in ordered mesoporous silica materials never stop growing since the Mobil Company’s first report in 1992 [1]. The first described synthetic procedures were largely optimised notably for working toward more sustainable processes. Nowadays, there is an increasing attention paid to the development of environmental-benign applications that employ as many renewable sources as possible while decreasing energy consumption. In this green context, it is of crucial importance to have a sustainable vision toward the entire process, from synthesis to applications. Consequently, recent efforts have been dedicated to the design of more environmentally friendly synthetic procedures [2,3]. The 12 principles of Green Chemistry first proposed by Anastas and Warner in 1998 suggest, among other things, the use of less hazardous chemicals and the design for energy efficiency [4].

The properties of porous silica (e.g., high surface area; high thermal, mechanical and chemical stability; and easy recovery) can be leveraged in a plethora of applications. As an example, the use of zeolites was extended over the years to include a broad window of possible applications, from ion exchange resin to catalysis, wastewater treatment and adsorption [5]. Mesoporous silica embedding trivalent or tetravalent cations (e.g., Al^3+^, Ga^3+^, Hf^4+^, Sn^4+^) can be employed for similar applications. Their larger pore size can extend even further the scope of these solids [6,7,8].

Within the large family of mesoporous materials, solids with a hexagonal arrangement of tubular pores (i.e., MCM-41 and SBA-15) are among the most widely studied. Their synthesis relies on the interaction of hydrolysed silica precursors with a pre-assembled micellar structure of surfactant molecules. To obtain a structured SiO_2_ network, a strong acidic or basic (depending on the targeted materials) medium is usually required. Indeed, close to neutral media, the hydrolysis of silica precursors is considerably slower as compared to condensation, and this might result in less-organized silica structures. However, the use of a strong acidic or basic medium is particularly eco-unfriendly, especially for large-scale production. Moreover, the traditional synthesis of both MCM-41 and SBA-15 materials requires the use of a hydrothermal treatment performed in a sealed autoclave at 100 °C or above, thus increasing the unfavourable ecological footprint. Different alternative approaches have been recently proposed. The rapid and room-temperature syntheses of MCM-41-like nanospheres were developed by Aprile et al. [9]. The particle size was decreased to the nanoscale (<100 nm) using specific conditions based on the dilute route reported by Cai et al. [10]. The same approach as the dilute route concept can be extended to SBA-15 solids. Moreover, the limited use of strong acids such as HCl could be considered as well. The generation of the acidic medium (prerequisite to the condensation of silica) can be obtained through the dissolution of the metal precursor (i.e., generally metal salts such as chloride or nitrate). This option was first proposed by Chen et al. [11], who used zirconium oxychloride for the formation of Zr-SBA-15. It was later optimised and adapted to the use of aluminium precursors and, more recently, to the synthesis of Al-Ga bifunctional silicates [12,13]. However, the hydrothermal step is still required in all the reported SBA-15 syntheses.

Among the different heteroatoms (with hetero being different from silicon) considered for the insertion within the SiO_2_ mesoporous network, aluminium and gallium were extensively investigated. Their strong activity in various catalytic reactions (e.g., acetylation [14,15,16], Friedel-Crafts alkylations [17,18], epoxidations [16], and Diels-Alder reactions [19,20]) was proven to be directly related to their insertion in the SiO_2_ matrix [13,14,21]. Going down the periodic table of elements, the insertion of indium was scarcely studied [22,23,24,25]. Moreover, to the best of our knowledge, all In-based silica materials reported so far were hydrothermally treated and synthesized under traditional conditions (i.e., strong acid/base medium) [26,27,28,29]. The reduced presence of In-SiO_2_ in the literature should not be attributed to a lack of interest but mainly to the intrinsic difficulties related to the isomorphic substitution of Si with In in the silica architecture. Indeed, the large size of this element could strongly impact the structural and textural properties of the materials and compromise its correct insertion. The presence of a substantial quantity of extra-framework indium species was detected in the materials prepared with a significant amount of indium as compared to Si (i.e., Si/In < 10) [27]. A deformation of the structured mesoporosity can be observed when employing cation with large ionic radii [13,30]. Indeed, to achieve a correct isomorphic substitution of framework Si atoms by trivalent cation, it is generally accepted (in the context of zeolites) that the X and Si should be of similar size (below 15% difference) and should have close electronegativity (maximum difference of 0.4 units on the Pauling scale) [31]. While indium fulfils the electronegativity criteria, it displays a significantly larger ionic radius. However, the limit was established for zeolite materials and previous results reported the correct insertion in the amorphous mesoporous structure of Sn and Hf, which are all out of limits [9,16,32]. The perspective of a silica-embedding indium as a single site is particularly interesting in several contexts. Indosilicates were already proven to be active in various catalytic processes in the gas phase [23,24,26] or as the dye removal compound [29].

In this study, we present a green strategy for the synthesis of indium-modified structured mesoporous silicas. Two sustainable procedures are proposed: the fast, room-temperature synthesis of extra-small nanoparticles and the acid-free synthesis of In-SBA-15. Hydrothermal treatment was avoided in both cases, thus decreasing the energy requirements and further improving the sustainability of the synthesis approaches. The insertion of In in the silica frameworks was successfully achieved while preserving the porosity and high specific surface area of the selected solids. The impact of the methods on the textural and structural features of the materials was investigated through an extensive characterization campaign.

## 2. Results and Discussion

### 2.1. Extra-Small Nanospheres (NS)

**Figure 1:**
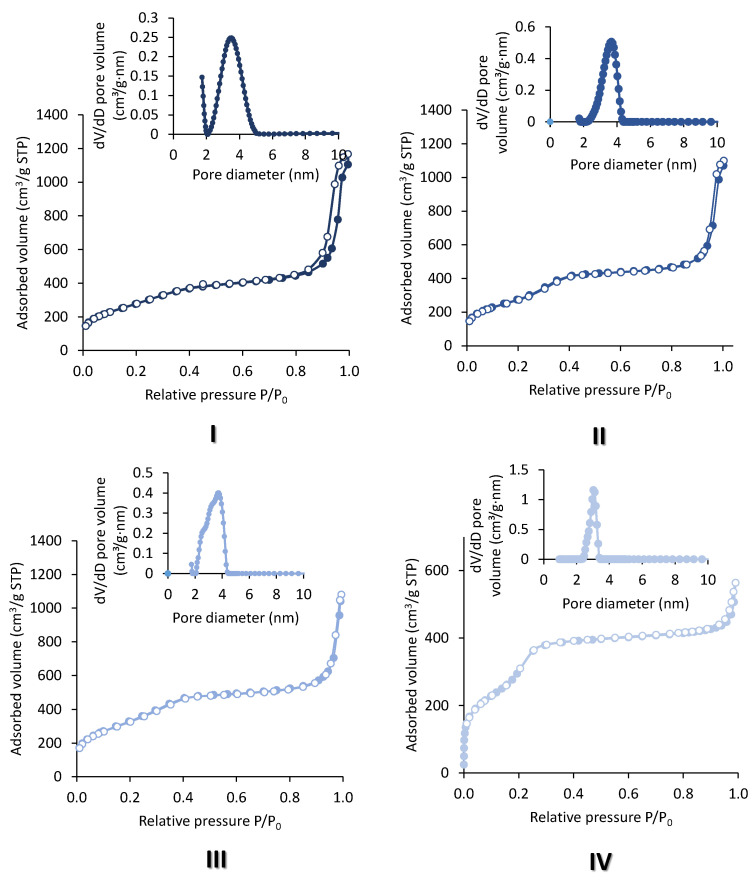
N_2_ physisorption adsorption (full symbols)-desorption (empty symbols) isotherms and pore size distribution of indium-based silica nanospheres. (**I**) In-NS-Nit, (**II**) In-NS-Cl-b, (**III**) In-NS-Cl-c, (**IV**) In-NS-Cl-d.

One of the key parameters for obtaining a successful insertion of heteroelements in the SiO_2_ network is represented by the precise control of the hydrolysis/condensation rate of the different precursors employed. Both inorganic precursors should display close rates to obtain a homogeneous distribution of the metal cations within the silica network, hence minimizing the presence of extra-framework species. Therefore, a preliminary study on the different indium precursors was performed. According to previous results on gallosilicate nanospheres [21], the investigations were first conducted using indium nitrate and selecting a Si/In ratio of 74. An aqueous solution of ammonia was used to obtain the required basic medium. These parameters were previously reported as particularly appropriate for the optimal insertion of metal cations within the silica matrix. Moreover, this specific synthetic procedure allowed forming extra-small particles (i.e., particle diameter below 50 nm with a narrow particle size distribution), which resulted in improved catalytic activity [9]. Indium nitrate was unsuccessfully dissolved in water or ethanol. However, to ensure optimal insertion, the miscibility of the precursor in the dilution medium is of crucial importance. Hence, to efficiently solubilize indium nitrate, an acidic medium was employed. The minimum quantity of HCl required to obtain a homogeneous solution was used (see the experimental for more details). This first sample was named In-NS-Nit. The material was extensively characterized, and the results are summarized in Table 1, Entry 1. The Si/In ratio assessed with ICP-OES measurement matches the targeted value. A Type IV adsorption-desorption isotherm typical of ordered mesoporous silica is displayed in Figure 1. The specific surface area is particularly high for mesoporous silica materials (above 1000 m^2^/g). As expected, the pore diameter is in the low window of mesopores and, therefore, the pore dimension was assessed via DFT calculations as this method is more suitable for pores below 4 nm than the traditional Barrett-Joyner-Halenda (BJH) method.

**Figure 2:**
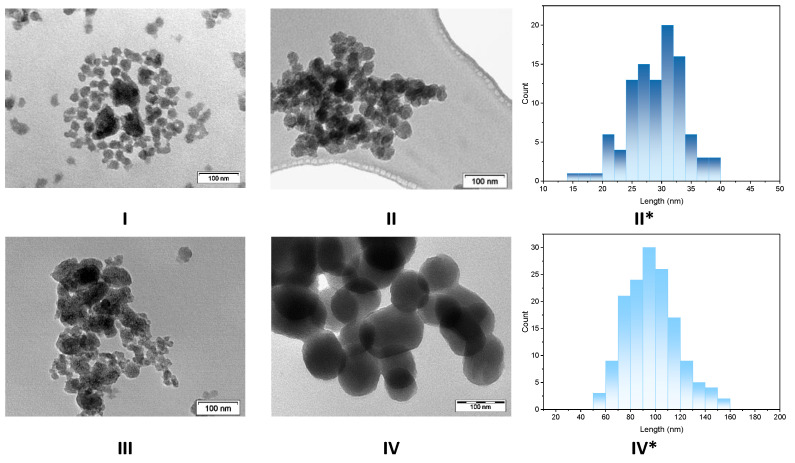
TEM images of extra-small nanospheres silica embedding indium and * particle size distribution assessed with TEM images and built upon analysis of 200 particles. The distribution was evaluated solely for spherical and homogeneous particles. (**I**) In-NS-Nit, (**II**,II*) In-NS-Cl-b, (**III**) In-NS-Cl-c, (**IV**,IV*) In-NS-Cl-d.

The morphology of the particles was inspected with Transmission Electron Microscopy (TEM). In-NS-Nit displays agglomerated particles of inhomogeneous shape (Figure 2I). Since it is known that the pH of the reaction medium is crucial for obtaining spherical, homogeneous, and small-sized particles, the perturbation of the morphology could be ascribed to the addition of the acidic solution (required to dissolve indium nitrate) to the reaction mixture. The presence of nitrate as counter ion could also impact the particle morphology. To identify the key parameter negatively influencing the morphology of the particles, two additional materials were synthesized. The first material was obtained employing indium chloride as the precursor (In-NS-Cl-a). This solid was synthesized following an identical procedure to that of the In-NS-Nit (i.e., dissolution of the precursor in acidified medium). The second synthesis was performed under similar conditions, but indium chloride was dissolved in absolute ethanol. The second solid was named In-NS-Cl-b. The characterization of both In-NS-Cl-a and -b with TEM (Figure 2II and Figure S1) revealed the presence of spherical particles of homogenous sizes (c.a. around 25 nm) solely for sample -b. Sample -a displayed a similar non-uniform particle morphology than In-NS-Nit, thus confirming that a minor variation of pH (i.e., around 0.3 pH unit) induced by the presence HCl 0.01 M is detrimental. The In-NS-Cl-b displayed the targeted morphological features, while the other textural parameters (i.e., SSA_BET_, DFT pore diameter, Si/In ratio) were similar to In-NS-Nit (see Table 1, Figure 2 and Figure S2). Considering these observations, indium chloride was selected as indium precursor for further investigations.

Three additional materials were synthetized to assess the impact of other parameters. The In-NS-Cl-c was prepared by employing a higher amount of indium (Si/In ratio of 37, entry 4 in Table 1). The In-NS-Cl-d was synthesized with a higher quantity of ammonia (Entry 5 in Table 1). Such an amount of ammonia is expected to enable the enlargement of the particle size to c.a. 100 nm. Finally, to study the influence of a closer contact of the two inorganic precursors on the insertion of In, the premixing of InCl_3_ and TEOS was considered for In-NS-Cl-e (Entry 6 in Table 1).

The specific surface areas of the newly synthetized materials were all above 1000 m^2^/g and were not impacted neither by the amount of indium, the amount of ammonia, nor by the pre-mixing of the precursors. All the samples displayed type IV isotherm, typical of ordered mesoporous materials (Figure 1). The shape of the isotherm corresponded to the adsorption of the monolayer-multilayer onto the silica surface. It was followed by capillary condensation, which occurs in the mesopores and gives rise to the small plateau observed at p/p^0^ close to 1. The interparticular void caused by the aggregation of the nanospheres is responsible for the steep increase at the end of the adsorption and is present in every sample. The specific surface area calculated based on the BET method was assessed in the linear section between p/p^0^ = 0.05 and p/p^0^ = 0.3, corresponding to the adsorption of the monolayer. The absence of hysteresis and the reversible nature of the isotherms are explained by the small width of the pores of the NS materials, which are below 4 nm and are attributed to the Type IVb isotherm shape according to the IUPAC denomination [33]. The pore size diameter was not influenced by the different parameters studied and it is situated in a narrow range from 3.5 to 3.7 nm. These values are in the low window of mesopores and were determined using DFT calculations. The only textural feature that was influenced by the different conditions was the total pore volume. Indeed, the sample In-NS-Cl-d showed a lower total pore volume (i.e., 0.9 cm^3^/g) as compared to the other samples (i.e., 1.5–2 cm^3^/g). This behaviour could be attributed to a combination of factors: (i) the larger size of particles induces a decrease of the interparticle void and (ii) under these reaction conditions, the pore size is lowered.

As expected, increasing the amount of ammonia from 1.0 g to 5.0 g leads to an increase in particle size, as shown on the TEM image displayed in Figure 2IV. The homogenous spherical shape of the particles was preserved, but their size was doubled, as shown in the particle size distribution (Figure 2II*,IV*). Finally, the pre-mixing of the two precursors gave rise to spherical particles of regular shapes. The TEM image of the In-NS-Cl-e is presented in the Supplementary Materials.

**Figure 3:**
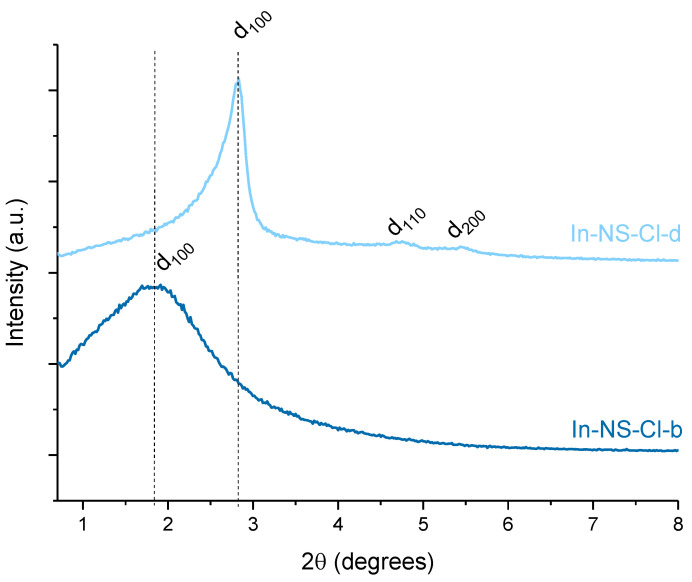
Small-angle XRD pattern of indium-based silica nanospheres.

The long-range order of the pores of In-NS-Cl-b and -d was assessed through small-angle XRD analysis. The XRD patterns of the remaining samples are presented in the Supplementary Materials (Figure S3) and displayed a similar aspect than In-NS-Cl-b. The patterns presented in Figure 3 display a broad signal ascribed to d_100_ reflection centred around 2θ = 1.8° and 2.8° for In-NS-Cl-b and -d, respectively. In-NS-Cl-b did not display the additional diffraction peak generally encountered in hexagonally arranged porous materials due to the extra-small particle size. On the contrary, the XRD pattern of In-NS-Cl-d showed the additional d_110_ and d_200_ reflection at respectively 2θ = 4.7° and 5.5°. The higher resolution of the XRD pattern can be explained by their larger particle size which improves the long-range order. For materials displaying a hexagonal array of mesopores such as silica nanospheres, the position of the d_100_ diffraction peak can be correlated to the lattice parameter, hence to the distance between the two atomic planes (see Supplementary Materials for more details). It is worth noting that, in the case of the In-NS-Cl-b, the broadness of the d_100_ contribution limits the use of Bragg law and, therefore, the quantitative evaluation of the lattice parameter. However, from a qualitative point of view, the shift at lower 2θ-degree values for the sample In-NS-Cl-b is clearly observable. This shift, coupled with the difference in pore diameter (0.7 nm), highlights a significative difference in wall thickness (WT) (i.e., WT_In-NS-Cl-b_ > WT _In-NS-Cl-d_). Therefore, the amount of indium trapped in the bulk of the SiO_2_ network is probably more important for the In-NS-Cl-b sample. This difference in wall thickness could be attributed to the concentration of ammonia. It was proposed [9,21] that the increase in ammonium hydroxide concentration promotes both the hydrolysis and condensation reactions of the inorganic precursors, with a preference for condensation. As a consequence, larger particles are formed with correspondingly thinner walls.

The amount of indium presents in the solids was determined using ICP-OES. Samples In-NS-Cl-b and -c displayed a Si/In ratio close to the theoretical ratio (77 and 33, respectively). However, increasing the amount of ammonia or pre-mixing the Si and In precursors influenced the quantity of indium. The experimental Si/In ratios for In-NS-Cl-d and -e were higher compared to the targeted ratio, which can be explained by an incomplete condensation of hydrolysed silica precursors due to the change of the pH of the solution.

The increased amount of indium (for In-NS-Cl-c) resulted in a loss of the spherical shape of the particles (Figure 2). Two hypotheses can be considered for the non-regular morphology of this solid: (i) the high amount of indium, which displays a large ionic radius, disturbed the silica network, or (ii) the high amount of InCl_3_ involved in the synthesis perturbed the pH of the medium, resulting in less-regular particles. To exclude one of the two hypotheses, an additional solid was prepared. A Si/In ratio of 74 was targeted and an amount of 280 µL of HCl 2M was added to mimic the quantity of HCl generated by the dissolution of the extra indium chloride needed for a Si/In ratio of 37. The characterization of this material (i.e., In-NS-Cl-f) is presented in the Supplementary Materials (Figure S5). The morphology, probed with TEM, is similar to In-NS-Cl-c with non-spherical particles of inhomogeneous shape. This confirms that the presence of HCl generated by the high amount of InCl_3_ causes the loss of morphology homogeneity. These observations are in agreement with the results reported above concerning the solubilization of the indium precursor in HCl 0.01 M. The pH measured for the solutions of In-NS-Nit, In-NS-Cl-a and In-NS-Cl-c were indeed close and smaller than the pH of the In-NS-Cl-b. Considering the outcomes of the parameters screening, a Si/In ratio of 74 combined with 1 g of ammonia and InCl_3_ as metal precursor are the optimal features for obtaining structured mesoporous materials and a correct insertion into the silica matrix.

The successful syntheses of indium nanospheres reported above give rise to a series of materials with the targeted amount of indium and interesting structural and morphological features. However, as mentioned previously, the challenge when dealing with heteroelements with an ionic radius significantly larger than Si is the possible formation of extra-framework species. The latter was previously described for other elements such as Ga and Hf [21,34]. The characterization of the chemical environment of indium was poorly reported in the literature. Motokura et al. [35] performed XPS investigations on indium supported on silica. They showed that the indium species loaded onto a alumino-silica surface displayed a binding energy shifted to higher value compared to In_2_O_3_, In(OH)_3_ and InCl_3_ (i.e., 446.5 ev vs. respectively 443.8 ev, 444.6 ev, 445.7 ev). All the NS were analysed through XPS. The results of the high-resolution spectra of the In 3d electrons were compared with the spectra of In_2_O_3_ as well as the one of a pristine NS impregnated with the indium species (NS-In-Impregnated). The latter solid was included to mimic the nature of the extra-framework indium cluster and their synthesis is reported in the SI. The In 3d region exhibits two contributions: 3d_5/2_ and 3d_3/2_. This doublet results from the spin−orbit splitting and the distance between the two peaks (i.e., 7.5 eV) was previously observed [35].

**Figure 4:**
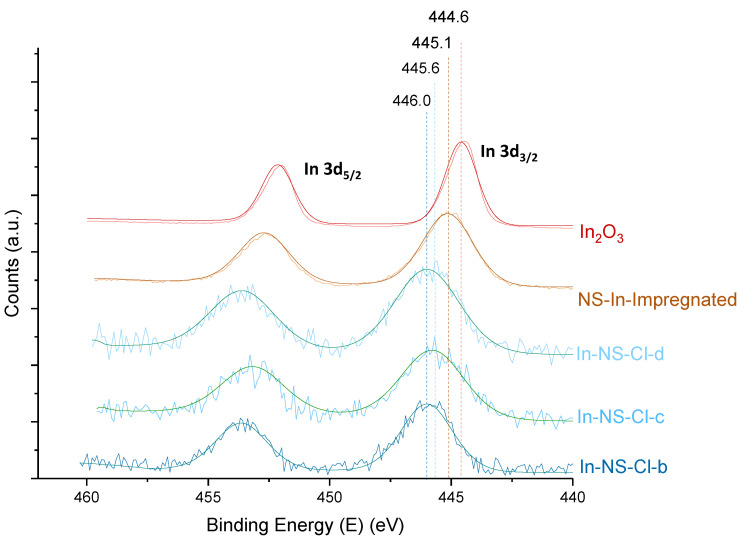
In 3d high resolution XPS pattern of extra-small silica nanospheres embedding indium, commercially available In_2_O_3_ and impregnated pristine SiO_2_ nanosphere. Pattern recorded for 100 scans at 25 keV as passing energy. The dotted lines are indicative of the maximum binding energy.

A clear shift toward higher binding energies can be observed for the materials newly synthesized in comparison with In_2_O_3_ (Figure 4). Moreover, the chemical environment of indium species in the samples differs from NS-In-Impregnated leading to the conclusion that in the In-NS samples indium is correctly inserted into the SiO_2_ matrix. The sample In-NS-Cl-c, which contains twice more In than In-NS-Cl-b and -d, displays a shift of binding energy toward In_2_O_3_, suggesting that part of the indium involved in the synthesis resulted in extra-framework species.

### 2.2. Acid-Free (AF) In-SBA-15

The synthesis of extra-small nanospheres results in considerable benefits for potential applications such as supports or catalysts. However, the narrowness of the pore diameter of the silica nanospheres can limit the use of such structures when dealing with larger molecules. SBA-15 has long been recognized for its excellent properties as a support as well as its enlarged pore size. Herein, the successful insertion of indium in SiO_2_ matrix proved with the investigation on silica nanospheres was extend to SBA-15 based solids. In the optic of sustainable syntheses of porous materials, the acid-free procedure previously reported for the use of zirconium was adapted to indium [11]. This synthesis is based on the acidity generated by the solubilization of the metal precursor. Based on the investigation on nanospheres, indium chloride was selected as metal precursor, a Si/In ratio of 74 was targeted and the material was named acid-free (AF) solids. The dissolution of indium chloride in the selected conditions induced a pH of about 3.5 pH unit, which is sufficient to promote the hydrolysis and condensation of silica oligomers and, therefore, the formation of structured materials. Chen et al. employed a hydrothermal treatment in a Teflon-sealed autoclave to finish the condensation of the materials. However, based on our knowledge of SiO_2_ materials, the use of a sealed autoclave and, more generally, the hydrothermal treatment, are not always mandatory to obtain a material with ordered architecture. To explore the possibility of performing a room-temperature synthesis of acid-free In-SBA-15, both hydrothermal and RT procedures were considered. It is known from the literature that an acidic medium could limit the insertion of elements in silica structure [34]. To overcome this issue, a pH adjusting step was considered. This additional step is usually performed using an aqueous ammonia solution (30%) to reach a pH = 9 after the hydrolysis/condensation of the Si precursor. This additional step allows terminating efficiently the condensation and it should also favour the incorporation of the heteroelements into the silica framework. To investigate this hypothesis, a pH adjusting step was performed on both the samples that were treated hydrothermally and the ones treated at room-temperature. Table 2 summarizes the different synthetic conditions employed for this second series of SBA-15-like materials.

As expected, the ICP-OES analysis performed on all materials revealed that the acidic medium decreased the quantity of indium present in the solids (entries 1 and 2). The pH adjusting step applied to samples In-AF-Cl-pH-HT and In-AF-Cl-pH-RT efficiently mitigated the indium insertion issue. Both solids displayed a Si/In ratio close to the targeted ratio. This result validates the need for the additional pH adjusting step and shows that the hydrothermal treatment does not influence the amount of indium in the solids.

**Figure 5:**
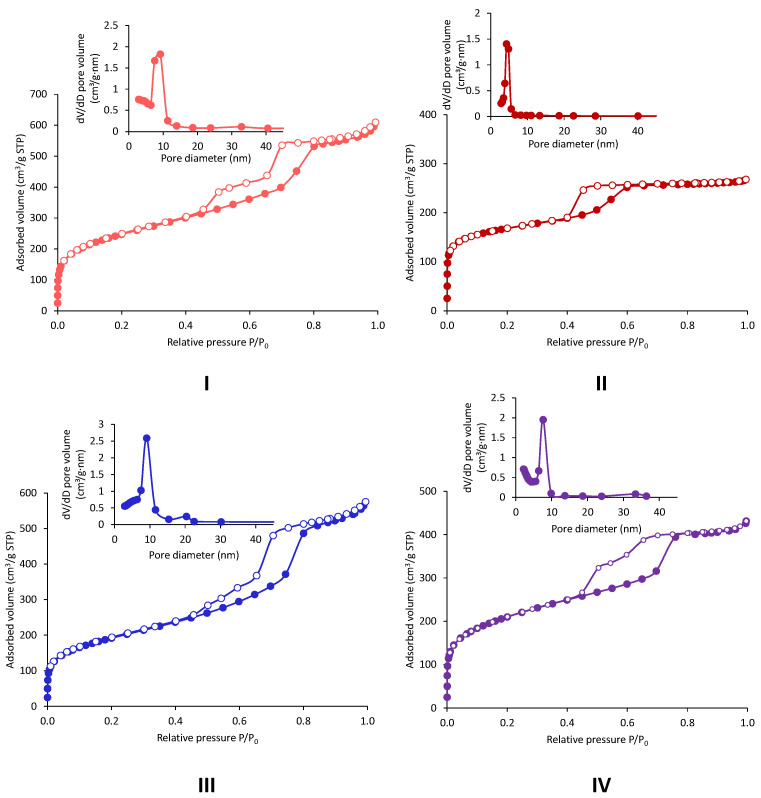
Adsorption (full symbols)-desorption (empty symbols) isotherms and pore size distribution of the indium-based silica mesoporous materials determined with N_2_ physisorption: (**I**)—In-AF-Cl-HT, (**II**)—In-AF-Cl-RT, (**III**)—In-AF-Cl-pH-HT, (**IV**)—In-AF-Cl-pH-RT.

The effect of the hydrothermal treatment and the pH adjustment step was particularly visible via an N_2_ physisorption analysis. The specific surface area of the In-AF-Cl-RT was 35% lower than the one of the thermally treated In-AF-Cl-HT sample, and the total pore volume of the former dropped down to 0.3 cm^3^/g as compared to 1.0 cm^3^/g (compare entries 1 and 2 in Table 2). The BJH pore diameter was also significantly decreased. Interestingly, when the pH was adjusted to 9, no more differences on the textural properties between samples with or without thermal treatment was observed (compare entries 3 and 4 in Table 2). The sample In-AF-Cl-pH-RT displayed a high specific surface area as well as a pore diameter and a pore volume close to the HT sample. This result indicates that the pH adjusting step can replace the hydrothermal treatment under static conditions, thus further enhancing the sustainability of the synthesis procedure. The N_2_ adsorption-desorption isotherms presented on Figure 5 display the type IV isotherm similarly to the one observed for the NS solids presented above. The plateau of the capillary condensation is more pronounced, especially for the In-AF-Cl-RT, which displays a smaller pore width. In contrast to the NS samples, hysteresis and type IVa isotherm are observed due to the larger pore diameter of SBA-15 solids (i.e., >4 nm). In-AF-Cl-RT displays a type H2 hysteresis as described by IUPAC, which is ascribed to the pore-blocking/percolation in pores of small dimension. The other samples show a type H5 hysteresis which are typical of porous structures containing both open and partially blocked mesopores. The hydrothermal treatment and/or the pH adjustment step can explain this hysteresis shape. The sharp nitrogen uptake visible at low p/p^0^ on all four isotherms indicates the presence of microporosity. The latter is more pronounced for the In-AF-Cl-RT as highlighted by the increased microporous volume (Figure 5).

**Figure 6:**
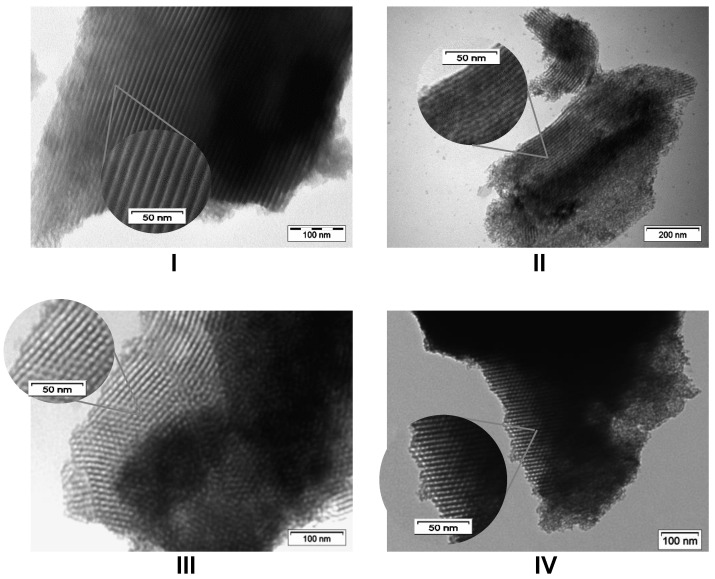
TEM images of acid-free In-SBA-15 solids: (**I**) In-AF-Cl-HT, (**II**) In-AF-Cl-RT, (**III**) In-AF-Cl-pH-HT and (**IV**) In-AF-Cl-pH-RT.

In terms of morphology, the series of In-AF materials clearly differ from the In-NS solids. Large particles of irregular shapes with well-defined hexagonal arrangements of pores, typical of SBA-15 architecture, are visible for all In-AF samples (Figure 6). Due to their inhomogeneous shape, their size was difficult to measure; hence, the particle size distribution was not evaluated. The TEM analysis also confirmed the smaller pores size and the larger ratio silica wall/porosity of In-AF-Cl-RT (see Figure 6II). The honey-comb structure clearly observable in both In-AF-Cl-pH-HT and In-AF-Cl-pH-RT solids testifies that the hydrothermal treatment is not required from a morphological point of view, and the pH adjustment step does not perturb the aspect of the particles.

**Figure 7:**
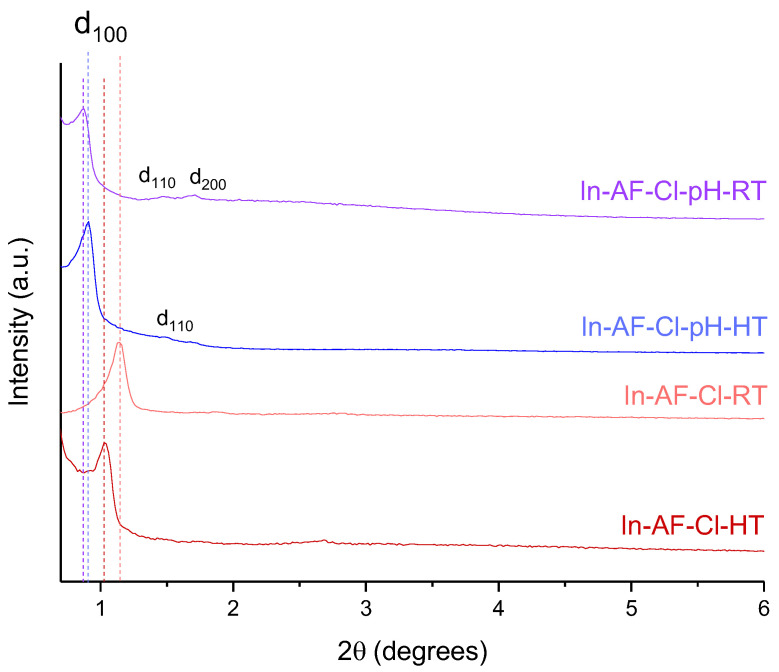
XRD small-angle pattern of indium-based silica materials. The line stands for the d_100_ diffraction peak.

As commented previously, the size of the SBA-15 particles was considerably larger compared to the NS samples (see TEM images). Therefore, the long-range order assessed by small-angle XRD pattern is significantly improved, as the d_100_diffraction peak f is more defined (Figure 7)_._ Moreover, d_110_ and d_200_ contributions are also visible for sample In-AF-Cl-pH-RT_._ The signals visible on the pattern (Figure 7) confirmed the hexagonal arrangement of pores typical of the SBA-15 structure. A clear shift of the position of the d_100_ diffraction peak was observed between the batches of samples prepared with and without pH treatment. The smaller value of 2θ for the samples obtained with the pH adjusting step results in a higher lattice parameter value and, therefore, in a larger distance between the two atomic planes. In the cases of In-AF-Cl-HT and In-AF-Cl-pH-HT, their BJH pore diameters are similar (see Table 2); therefore, in this case the pH treatment resulted in a solid with thicker silica walls (see Supplementary Materials for more details).

**Figure 8:**
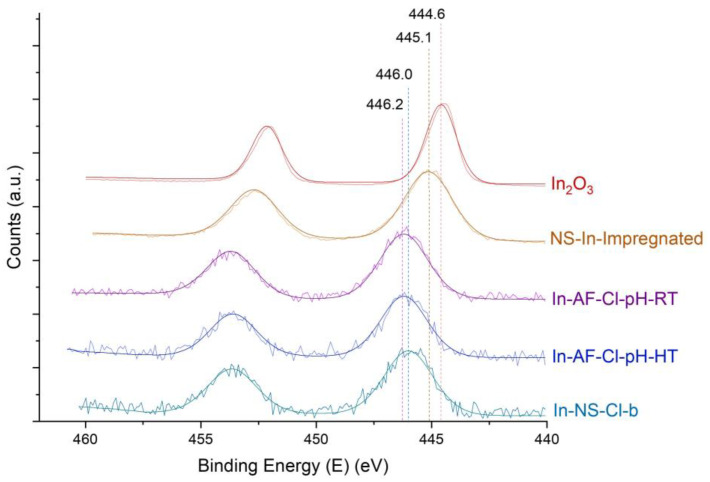
In 3d high resolution XPS pattern of indium acid-free SBA-15, commercially available In_2_O_3_ and impregnated pristine SiO_2_ nanosphere. Pattern recorded for 100 scans at 25 keV as passing energy. The dotted lines are indicative of the maximum binding energies.

Finally, similarly to the NS samples, the In-AF-Cl-pH-RT and In-AF-Cl-pH-HT were analysed using XPS to assess the insertion of indium inside the SiO_2_ matrix (Figure 8). Their spectra were also compared to In_2_O_3_, In-SiO_2_-impregnated samples and the In-NS-Cl-b (i.e., the best representative material of the series of nanospheres with an Si/In ratio of 74 and extra-small particle size). The comparison between the two AF samples and the NS sample shows that the binding energies of “acid-free” materials are slightly higher (446.2 vs. 446.0). The reported values are the averages over three different spots from the same sample. The shift can be explained by a slightly better insertion in the conditions employed for “acid-free” SBA-15 synthesis accompanied by a pH treatment step. 

## 3. Materials and Methods

### 3.1. Materials

Cetyltrimethylammonium (CTAB) and Tetraethoxysilane (TEOS, 97%) were purchased from TCI (Oakland, CA, USA); Ammonia (aq. 30%) an HCl 2 M from Carl Roth (Karlsruhe, Germany); Pluronic 123 from Sigma-Aldrich (St. Louis, MO, USA); and In(NO_3_)_3_·xH_2_O and InCl_3_·xH_2_O from Alfa Aesar (Haverhill, MA, USA). The absolute ethanol came from Fisher Scientific (Waltham, MA, USA) (analytical grade).

### 3.2. Catalyst Synthesis

#### 3.2.1. Extra-Small Silica Nanospheres

The synthesis procedure was adapted from Maertens et al. [21] for the use of indium. The surfactant, CTAB (656.0 mg, 1.800 mmol), was dissolved in milli-Q water (314 mL, 17.4 mol) in a 1L polypropylene bottle. A solution of 30% ammonia (1.12 mL, 8.6 mmol) was added dropwise to the clear solution. The solution was stirred at room temperature for 30 min (800 rpm). The In precursors (In(NO_3_)_3_·xH_2_O or InCl_3_·xH_2_O depending on the material) were dissolved at the desired Si/In ratio with sonication in their respective ideal dilution medium. The In precursor and TEOS (3.000 g, 13.97 mmol) were added simultaneously in a dropwise manner to the basic medium. The solution was stirred for 30 min, then filtered and washed three times with distilled water and EtOH alternately

Molar ratios: 1.000 TEOS:0.1288 CTAB:0.61 NH_4_OH:1.25 10^3^ H_2_O:x In(NO_3_)_3_·xH_2_O/InCl_3_·xH_2_O

#### 3.2.2. Acid-Free SBA-15 Particles

The synthesis protocol was inspired by Chen et al. [11] and adapted for the use of indium. In a 500 mL polypropylene bottle, P123 (1.74 g, 0.300 mmol) was dissolved in 162 mL of milli-Q water. After the complete dissolution of the surfactant, the indium precursor, In(NO_3_)_3_·xH_2_O (310 mg, 0.87 mmol) or InCl_3_·xH_2_O (257 mg, 0.87 mmol) was added to the medium. The solution was stirred for 30 min before the addition of TEOS (13.80 g, 64.36 mmol), then was stored for four hours at room temperature. The bottle was placed in a water bath heated at 40 °C and stirred overnight. The batch was then separated into equal parts; one was placed in an oven for static hydrothermal treatment (24 h, 100 °C) and the other was stirred at room temperature for 24 h. At the end of the day of treatment, the two batches were again divided into equal parts. One was filtered and washed three times with distilled water. The second batch was treated with NH_3_ (aq, 30%) until a pH of 9 was reached (control with pH paper). The solutions were stirred at room temperature for 24 h before filtration and washing.

Molar ratio: 1.00 TEOS:4.66 10^−3^ P123:140 H_2_O:0.0135 In(NO_3_)_3_·xH_2_O/InCl_3_·xH_2_O

All solids were dried overnight at 60 °C and calcined in air at 550 °C for 5 h (heating/cooling rate: 2 °C min^−1^) to remove the surfactant.

#### 3.2.3. Characterization

Nitrogen adsorption-desorption analyses were carried out at 77 K using a Micromeritics ASAP 2420 volumetric adsorption device. The degas treatment was applied to the sample before the analysis for 8 h at 150 °C under reduced pressure (0.1 mbar). To assess the surface area, the Brunauer–Emmet–Teller (BET) method was applied in the range of 0.05–0.30 P/P_0_. The pore size distributions were determined from the adsorption isotherm using the Density Functional Theory method with cylinder geometry and the N_2_-Cylindrical Pores-Oxide surface model for the nanospheres. For the SBA-15 samples, the Barrett–Joyner–Halenda (BJH) method was employed with the Kruk-Jaroniec-Sayari (KJS) correction. Transmission electron microscopy (TEM) images were captured using a Philips Tecnai 10 microscope operating at 80 kV. The chemical composition of the sample was determined by Inductively Coupled Plasma Optical Emission Spectroscopy (ICP-OES) with an Optima 8000 Spectrometer. Powder X-ray diffraction (XRD) patterns were recorded on a PANalytical X’pert diffractometer with Cu Kα radiation (k = 1.54178 Å). The X-ray photoelectron spectra were recorded on a ThermoFisher ESCALAB 250Xi spectrometer operating with a monochromatic Al Ka X-ray source (1486.6 eV) and a hemispherical deflector analyser (SDA) working at constant pass energy (CAE). The diameter of the X-ray spot was set to 300 µm. The base pressure of the analyser chamber amounted to 2 × 10^−8^ Pa. A flood gun with electrons and Ar ions was used for the compensation of the charges induced by photoemission. Survey spectra and high-resolution spectra were recorded as having a pass energy of respectively 150 eV and 25 eV; 100 scans were recorded for the indium signal. The chemical shift was shifted with respect to C 1s (C-C bonding) signal used as reference and fixed to 284.4 ev. The chemical components on the photoemission spectra were fitted with a Gaussian-Lorentzian line shape. The Gaussian-Lorentzian (L/G) ratio, full-width at half maximum (FWHM), kinetic energy and intensity (peak area) were adjusted during the procedure.

## 4. Conclusions

The synthesis of two series of In-modified mesoporous silica materials was successfully achieved. The sustainability of the synthesis methods was enhanced in different ways. Extra-small SiO_2_ nanospheres were obtained via a rapid procedure under dilute NH_3(aq)_ medium at room temperature. SBA-15-like structures were obtained by generating the acidity in situ via the solubilization of the indium precursor in the medium. Several parameters and their impacts on the structural and morphological properties as well as on the insertion of indium were evaluated. In the case of the silica nanospheres, the most suitable set of parameters (e.g., 1.0 g of NH_3_ (aq), Si/In of 74 with InCl_3_ as precursor) allowed obtaining high specific surface area solids with spherical shapes and displaying an amount of In inserted in the SiO_2_ framework close to the nominal value. For the AF samples, the pH adjusting step was required to favor the insertion of indium within the SiO_2_ network. High specific surface area and large pores were obtained. The insertion of indium was probed using XPS. The binding energy shift of the newly synthesized materials compared to both In_2_O_3_ and impregnated NS sample confirmed the correct isomorphic substitution of Si with In in the SiO_2_ network. In all cases, the best materials were obtained without hydrothermal treatment under static conditions, thus enhancing further the sustainability of the synthesis approaches.

## Figures and Tables

**Table 1:** Synthetic conditions and physico-chemical properties of the silica nanospheres embedding indium MPD: mean pore diameter determined via DFT for NS samples. n.a.: not applicable.

Entry	Sample	Amount of Ammonia	Medium of Precursor Solubilization	Si/In Ratio (ICP-OES)		SBET (m^2^/g)	Pore Diameter (nm)	Total Pore Volume (cm^3^/g)	Particle Size (nm)
				Targeted	Exp.				
1	In-NS-Nit	1.0 g	Acidic	74	71	1033	3.5	1.8	n.a.
2	In-NS-Cl-a	1.0 g	Acidic	74	73	1073	3.5	2.1	n.a.
3	In-NS-Cl-b	1.0 g	Alcohol	74	77	1033	3.7	1.5	29
4	In-NS-Cl-c	1.0 g	Alcohol	37	33	1256	3.7	1.7	n.a.
5	In-NS-Cl-d	5.0 g	Alcohol	74	55	1250	3.0	0.9	97
6	In-NS-Cl-e	1.0 g	Alcohol	74	55	1123	3.4	1.9	n.a.

**Table 2:** Synthetic conditions and physico-chemical properties of silica mesoporous materials embedding indium, synthesized through an acid-free process.

Entry	Sample	Precursor	Hydrothermal Treatment	pH Adjustement	Si/In Ratio (ICP-OES)	S_BET_ (m^2^/g)	BJH Pore Diameter (nm)	Pore Volume * (cm^3^/g)
1	In-AF-Cl-HT	InCl_3_	**✓**	**✗**	505	873	9	1.0 (0.02)
2	In-AF-Cl-RT	InCl_3_	**✗**	**✗**	586	565	4	0.3 (0.1)
3	In-AF-Cl-pH-HT	InCl_3_	**✓**	**✓**	72	678	9	0.7 (0.02)
4	In-AF-Cl-pH-RT	InCl_3_	**✗**	**✓**	85	734	8	0.9 (0.05)

* Microporous volume in parenthesis calculated according to t-plot between 3 and 5 Å of thickness.

## Data Availability

The data presented in this study are available on request from the corresponding author.

## Supplementary Materials

The following supporting information can be downloaded at https://www.mdpi.com/article/10.3390/molecules29010102/s1: Figure S1: TEM image of two silica nanospheres embedded indium; Figure S2: N2 physisorption adsorption-desorption isotherm and pore size distribution of two silica nanospheres embedding indium; Figure S3: Small angle XRD diffraction pattern of silica nanospheres samples embedding indium; Figure S4: N_2_ physisorption adsorption-desorption isotherm and TEM image of In-NS-Cl-f; Table S1: XRD parameters for silica nanospheres and acid-free SBA-15 samples 2θ is the diffraction angle of the d_100_ diffraction peak and a is the lattice parameter; and Figure S5: Bidimensional hexagonal lattice in which d_100_ is the inter-reticular distance and a0 is the lattice parameter.
