# Supplementary material for: Indium-Based Silica Materials: Sustainable Syntheses Combined with a Challenging Insertion in SiO2 Mesoporous Structures
Source: Molecules. 2023 Dec 22;29(1):102. doi: 10.3390/molecules29010102 (PMC10779520; doi:10.3390/molecules29010102)
Supplement: Supplementary file 1 [file molecules-29-00102-s001.zip › molecules-2735720-supplementary.pdf]

Supporting information

Indium-based silica materials: sustainable syntheses combined with a challenging insertion in SiO_2 mesoporous structures.

Amélie Maertens and Carmela Aprile

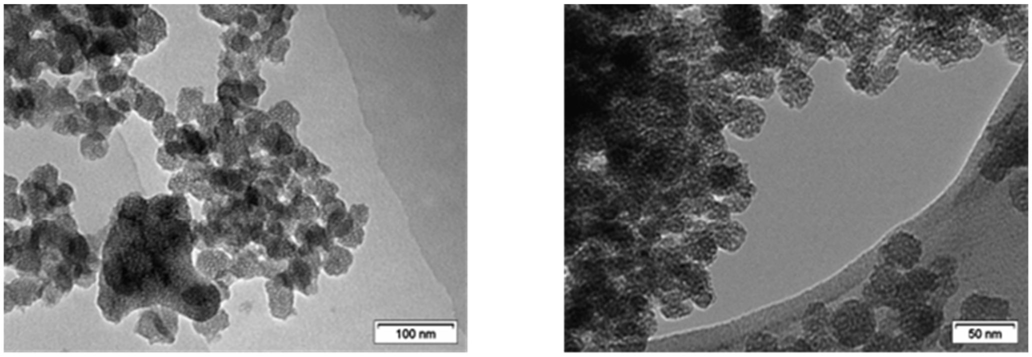

In-NS-Cl-a In-NS-Cl-e

Figure S1. TEM image of two silica nanospheres embedded indium

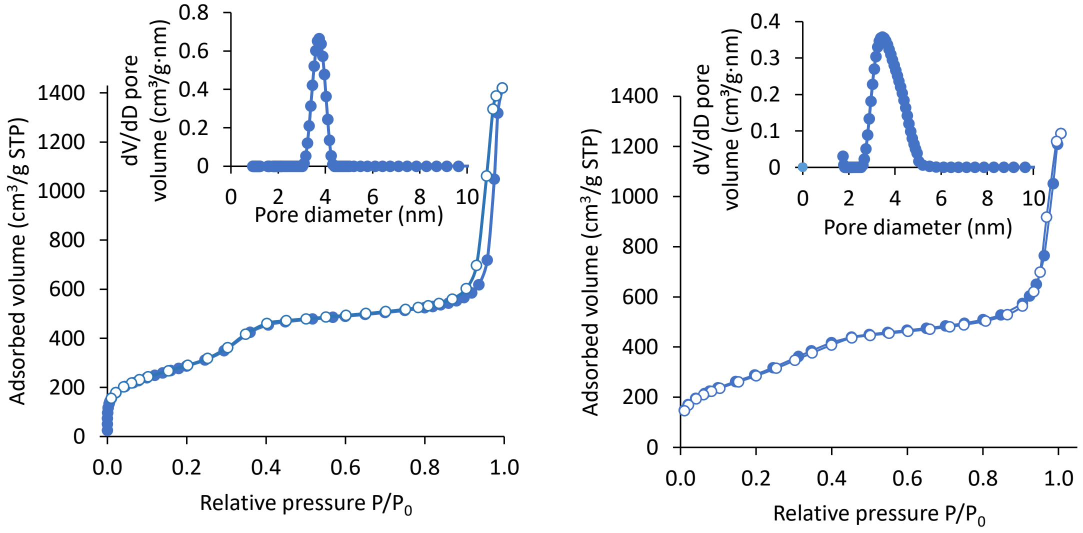

In-NS-Cl-a In-NS-Cl-e

Figure S2. N_2 physisorption adsorption-desorption isotherm and pore size distribution of two silica nanospheres embedding indium.

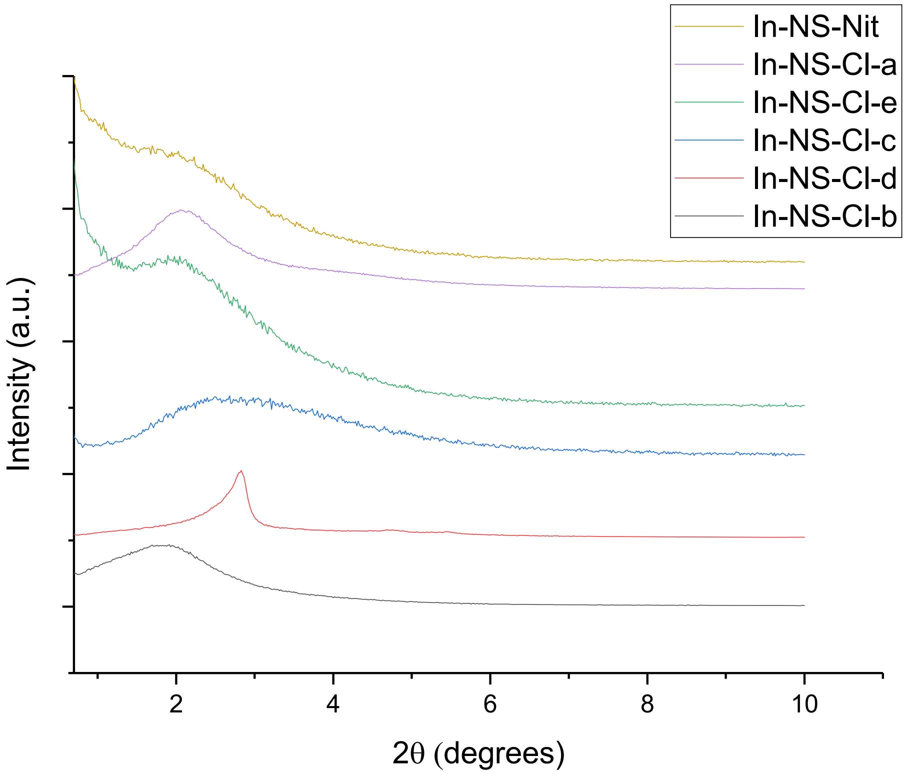

Figure S3. Small angle XRD diffraction pattern of silica nanospheres samples embedding indium

Table S1. XRD parameters for silica nanospheres and acid-free SBA-15 sample. 2ϑ is the diffraction angle of d_{100} diffraction peak, a is the lattice parameter.

Samples	2θ	d_{100} (nm)	a (nm)	Wall thickness (nm)
**$NS-Cl_3$ (a)**	≈1.8	-	-	-
**$NS-Cl_3$ (b)**	≈2.8	-	-	-
**$NS-Cl_3$ (c)**	≈2.1	-	-	-
**$NS-Cl_3$ (d)**	2.1	4.2	4.9	1.5
**$NS-Cl_3$ (e)**	≈2.0	-	-	-
**$NS-NO_3$ (a)**	≈2.3	-	-	-
**$AF-Cl_3$-HT**	1.0	8.7	10.0	1.0
**$AF-Cl_3$-RT**	1.1	7.7	8.9	4.9
**$AF-Cl_3$-pH_HT**	0.9	9.7	11.2	2.2
**$AF-Cl_3$-pH_RT**	0.9	10.1	11.7	3.7

d_{100} is calculated as follow : $\frac{0.154}{2\sin\theta}$, a as :$\frac{2d_{100}}{\sqrt{3}}$ and wall thickness : $a - BJH/DFT\ pore\ diameter$

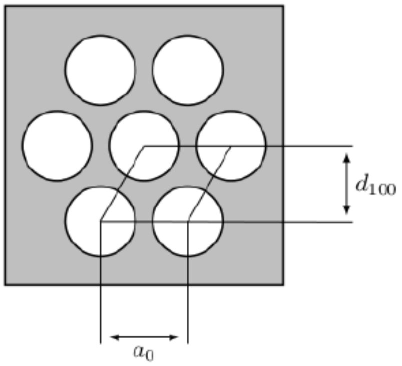

Figure S4. Bidimensional hexagonal lattice. d_{100}, inter-reticular distance and a_0, lattice parameter.

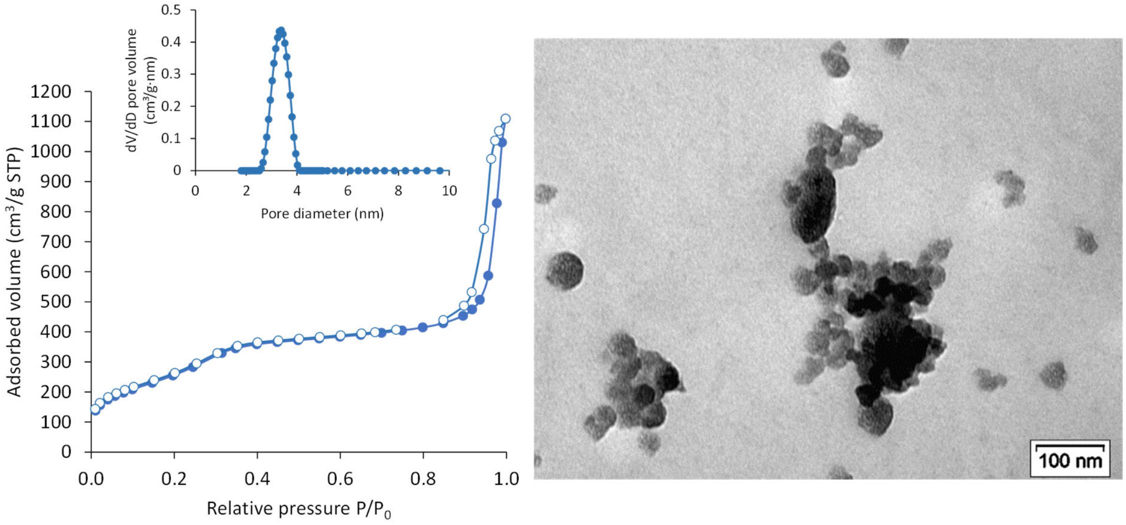

Figure S5. N_2 physisorption adsorption-desorption isotherm and TEM image of In-NS-Cl-f
